# Development of a novel self-sanitizing mask prototype to combat the spread of infectious disease and reduce unnecessary waste

**DOI:** 10.1038/s41598-021-97357-6

**Published:** 2021-09-14

**Authors:** Matthew J. Crawford, Sepehr Ramezani, Roghaie Jabbari, Pawan Pathak, Hyoung J. Cho, Brian N. Kim, Hwan Choi

**Affiliations:** 1 Department of Biomedical Sciences, University of Central Florida, Orlando, 32816 USA; 2 Department of Mechanical and Aerospace Engineering, University of Central Florida, Orlando, 32816 USA; 3 Department of Electrical and Computer Engineering, University of Central Florida, Orlando, 32816 USA; 4 Tehran, Iran

**Keywords:** Biomedical engineering, Mechanical engineering

## Abstract

With the spread of COVID-19, significant emphasis has been placed on mitigation techniques such as mask wearing to slow infectious disease transmission. Widespread use of face coverings has revealed challenges such as mask contamination and waste, presenting an opportunity to improve the current technologies. In response, we have developed the Auto-sanitizing Retractable Mask Optimized for Reusability (ARMOR). ARMOR is a novel, reusable face covering that can be quickly disinfected using an array of ultraviolet C lamps contained within a wearable case. A nanomembrane UVC sensor was used to quantify the intensity of germicidal radiation at 18 different locations on the face covering and determine the necessary exposure time to inactivate SARS-CoV-2 in addition to other viruses and bacteria. After experimentation, it was found that ARMOR successfully provided germicidal radiation to all areas of the mask and will inactivate SARS-CoV-2 in approximately 180 s, H1N1 Influenza in 130 s, and *Mycobacterium tuberculosis* in 113 s, proving that this design is effective at eliminating a variety of pathogens and can serve as an alternative to traditional waste-producing disposable face masks. The accessibility, ease of use, and speed of sanitization supports the wide application of ARMOR in both clinical and public settings.

## Introduction

Since being first reported in December 2019, coronavirus disease 2019 (COVID-19) has continued to spread worldwide with more than 164 million confirmed cases and 3.4 million related deaths as of May 20, 2021^1^. Severe acute respiratory syndrome coronavirus 2 (SARS-CoV-2) is the beta coronavirus which causes COVID-19, and is able to be spread via multiple modes of transmission, including direct contact and through airborne particulates^2^. As a result, the importance of methods to reduce the spread of all types of infectious disease between individuals has been emphasized. While mask wearing as a mitigation technique has been effective, it has also revealed the shortcomings of current mask technologies.

Previous studies have shown masks to be effective at blocking the release of respiratory particles into the wearer’s nearby environment, while also acting as a filter that can reduce the exposure to these infectious droplets^3,4^. However, the ability of a mask to protect the wearer is reduced when it is not used properly. One of the most common faults in the use of a protective face covering is contamination. Improper handling and storage of a mask when not in use, as well as prolonged use without sanitization, and physical touching of the mask material with infected hands can all contribute to the contamination of the face covering and the exposure of the wearer to these pathogens^5^.

Current mask technology is limited to two broad categories of face coverings: disposable and reusable. Both types have unique advantages and disadvantages, allowing for significant room for improvement. Disposable masks are designed for single-use and can easily be thrown away and replaced when contaminated, making them useful in clinical settings. However, the United Nations reported in July 2020 that an estimated 75% of the waste from disposable masks will end up in landfills or in the oceans^6^. A September 2020 study estimated that 16,659 tons of medical waste is produced daily solely in Asia^7^. With the spread of the COVID-19 pandemic, the rate of production of medical waste in the United states has increased from 5 million tons/year to 30 million tons/year^8^. It is estimated that 75–90% of this waste is composed of nonhazardous paper and plastic materials, which includes disposable face masks and other personal protective equipment (PPE)^9^. Furthermore, the World Health Organization expects the demand for disposable PPE to increase by up to 20% by 2025 as additional emphasis is placed on reducing the spread of infectious disease^10^.

Reusable masks significantly reduce the amount of plastic waste produced; however, the repeated use of a single face covering can lead to the accumulation of harmful viruses and bacteria on the protective material^11^. In order to maintain the function of reusable face coverings, they must be constantly washed, resulting in the expenditure of large amounts of water and electricity, while also taking extended periods of time for the sanitization process to be completed, making them expensive and impractical in clinical settings.

When considering alternative methods to sanitize a face covering, few options match effectiveness with practicality. Antiseptic solutions such as bleach are primarily used to disinfect hard surfaces and require copious amounts of water when used on porous materials^12^. Autoclaves are commonly used to sterilize laboratory materials; however, these machines are expensive and impractical for use in many clinical settings and by the public^13^. One method that combines efficiency and functionality is the use of concentrated ultraviolet radiation to sanitize surfaces^14–16^. Ultraviolet C (UVC) radiation has been directly tested on SARS-CoV-2, and has been shown to hinder viral replication by damaging the nucleic acid genetic material^15^. It was found that 254 nm UVC at an energy dosage of 5 mJ/cm^2^ inactivates 99% of the virus on surfaces^15^. Buonanno et. al. showed the effectiveness of using ultraviolet C radiation at wavelength 222 nm to destroy the outer shell of coronaviruses similar to SARS-CoV-2^16^. It was found that an energy dosage of 2 mJ/cm^2^ successfully inactivated 99.9% of the alpha coronavirus HCoV-229E and 99.99% of the beta coronavirus HCoV-OC43^16^. Germicidal UV light has long been proven useful in disinfecting surfaces to reduce the spread of other pathogens such as *Mycobacterium tuberculosis*, H1N1 Influenza, and *Escherichia coli*^14,16,17^.

When quantifying UV light, a measure of intensity is often used. Light intensity is the amount of energy given off to a specific area over some period of time. Therefore, a higher light intensity means that energy is being provided to a specific area at a higher rate. UV light is also often described in terms of wavelength of excitation. Shorter wavelength light has a higher excitation energy; therefore, a shorter wavelength lamp will give off a greater amount of energy than a longer wavelength lamp of the same intensity over the same period. Commonly used wavelengths of germicidal UVC are 270 nm, 254 nm, and 222 nm. Although the 254 nm and 222 nm lamps may give off slightly more excitation energy, the light sources for these wavelengths are much larger and heavier compared to the commercially available 270 nm-producing LED lamps, which are quite small and comparatively lightweight^14^.

There are currently products on the market that utilize UVC as a germicidal method for face masks, however each of these products have distinct disadvantages compared to the design we propose. Some products combine a UVC chamber with a filtering face covering, in which the air is exposed to UV light as it is breathed in by the wearer^18,19^. The manufacturer claims a 99.93% effectiveness rate against the two pathogens that were tested: *E. coli* and *Staphylococcus*, both of which are very UV-susceptible^18^. This design could have potential drawbacks against more UV-resilient pathogens, such as SARS-CoV-2. The rate of airflow cannot be manually adjusted, as it is dependent on the rate of breathing of the wearer. As a result, the time pathogens are exposed to the UV remains constant, which could lead to decreased effectiveness against viruses that take larger dosages of UV light to become inactivated. Other products feature a sanitization chamber that provides UV exposure directly to the face covering, however the decreased portability of these devices negatively impacts convenience and accessibility in both clinical and on-the-go settings^20^.

Given the importance of facial coverings in order to reduce the transmission of COVID-19 between individuals, insufficient attention is given to the improvement of current mask design function and sustainability. There is a need for a mask that is more easily sanitized and can still be easily put on and removed, while also promoting healthy mask wearing. As a result, we have developed the Auto-sanitizing Retractable Mask Optimized for Reusability (ARMOR), to further combat the spread of COVID-19 and other infectious diseases and reduce unnecessary waste.

## ARMOR prototype design

**Figure 1:**
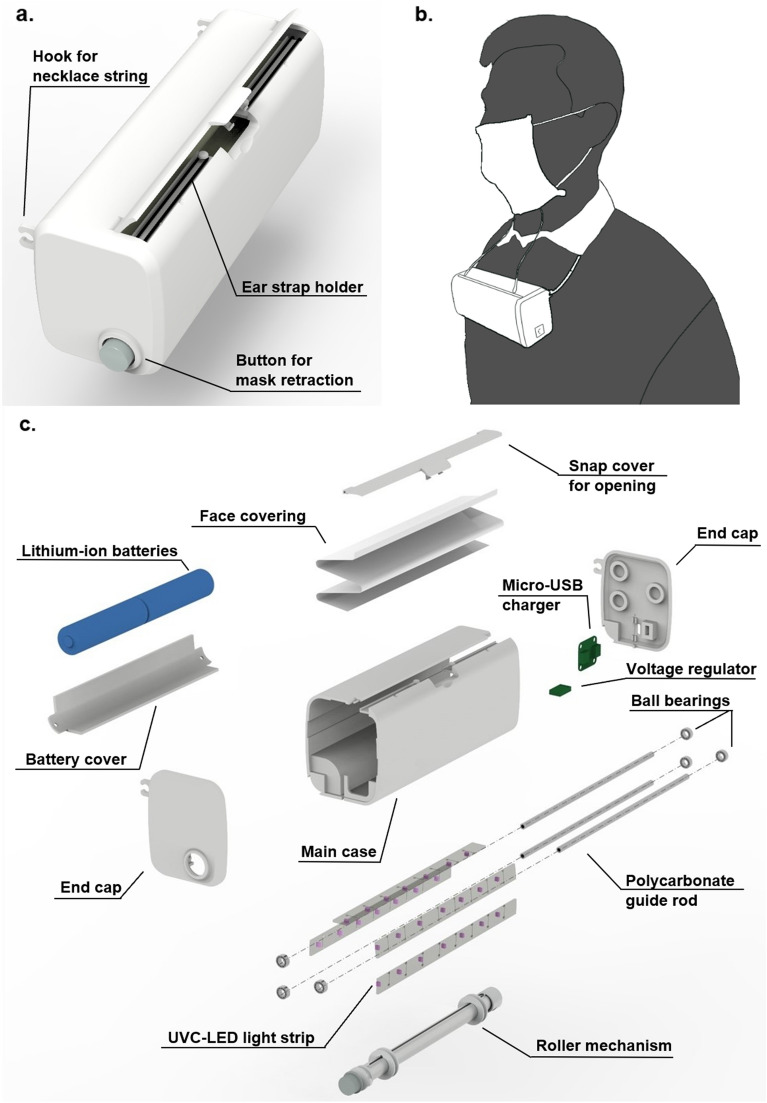
ARMOR exterior components (**a**), prototype demonstration (**b)**, and interior components (**c**). Individual exterior and interior components are displayed as shown in a 3D design software and are labeled to present all features of ARMOR. Interior components are shown in exploded view.

The idea behind ARMOR is simple: use ultraviolet C radiation to kill bacteria and viruses on the face covering. However, there was much to be considered in the design. First, in order to provide maximum UVC exposure to the mask and limit exposure to the wearer, the light-emitting diode (LED) lamps must be contained within a case with the face covering at the time of sterilization. To achieve this, a case was designed to be worn around the wearer’s neck, with the mask contained inside while not being used. A snap-in-place cover is used to close off the opening at the top of the case in order to reduce the risk of UV exposure and prevent dust from entering the sanitization chamber when the mask is inside (Fig. 1a). Underneath the cover is a tray with two hooks designed to hold the ear loops in place while the mask is in the case. When the user requires use of the face covering, the cover can be opened, deploying the ear straps of the face covering for the user to grab so it can be safely and easily extracted from the case without contamination and worn for as long as necessary (Fig. 1b). If the face covering becomes contaminated, it can be retracted back into the case with a simple press of a button, the ear straps can be hooked back in place, and it is then disinfected.

The penetration depth of UV light in most polymers is between 0.025 and 0.05 mm^21^. In order to protect the wearer against unwanted UV exposure, the thickness of the case is 2 mm in all areas except the grooves where the UV lamps reside, where it is 1.3 mm thick. This prevents UV penetration through the case and eliminates exposure to the wearer. The interior of ARMOR was designed to promote ease of use and maxize UVC exposure to all parts of the face covering (Fig. 1c). In this initial prototype, a cotton cloth was used as a sample face covering. Rods were used to define the track the mask follows when inside the case, creating a zig-zag pattern that prevents the material from folding in on itself and blocking areas from the LEDs. Polycarbonate was the selected material for these rods because it is permeable to UVC, allowing the germicidal radiation to reach the areas of the mask in direct contact with the rods. Ball bearings allow the rods to rotate while the mask glides over the track. A roller mechanism was employed to retract and deploy the face covering. One side of the roller contains an anchor which attaches to the left end cap, and the other side of the roller contains a ratchet mechanism which slips when the mask is being extracted. As the mask is pulled from the case, two strings attached to the bottom of the mask cause the roller to rotate, adding tension to a spring inside the roller. Upon complete extraction, the ratchet mechanism holds the spring tension until the retractor button is pressed, causing the clutch to disengage, allowing the spring tension to be released, which pulls the mask back into the case. The guides surrounding the roller act as a spool for the incoming string.

**Figure 2:**
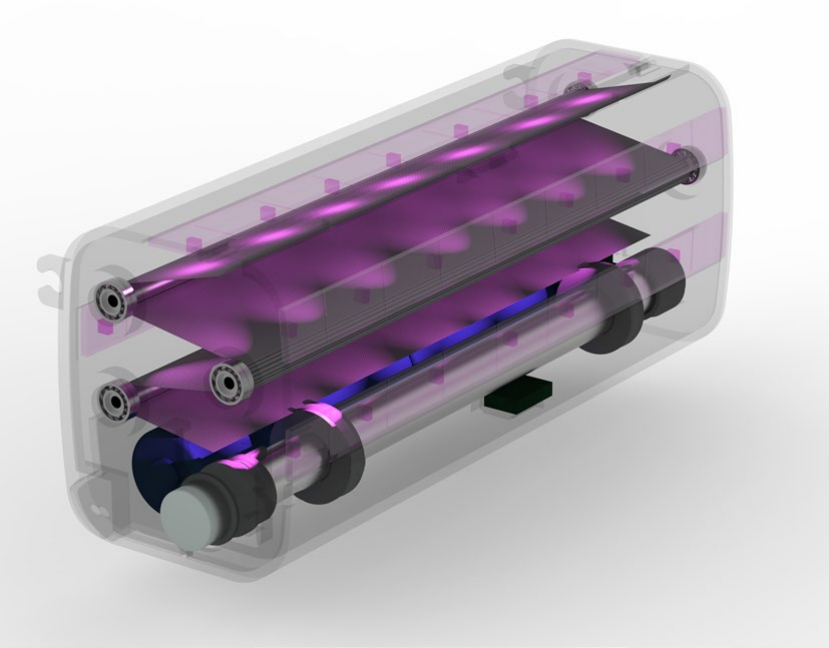
Schematic of UVC light exposure on the face covering inside ARMOR.

Four strips of seven 270 nm UVC-producing LEDs (28 lamps total) (cleanUV™, Waveform Lighting, Vancouver, WA) were placed in different locations around the interior of the case to provide exposure to all surfaces of the face covering (Fig. 2). Two rechargeable 3.7 V lithium-ion batteries (lithium-ion cylindrical battery, Adafruit, New York City, NY, USA) serve as the power source for ARMOR. The LEDs require a 12 V power source, therefore two isolated step-up voltage regulators (step-up regulator, Pololu Robotics and Electronics, Las Vegas, NV, USA) were used to generate a 12 V output. The batteries are confined in a separate space from the mask in ARMOR and can easily be accessed and removed for recharging with the removal of the rear battery cover. A push-button switch (push-button switch 1A, CW Industries, Southampton, PA, USA) allows for the LEDs to be turned on and off. The current iteration features a micro-USB port for recharging the batteries to provide increased ease of use (micro-LiPo charger with micro-USB jack, Adafruit, New York City, NY, USA). The total combined weight of the device including the rechargeable batteries is 242.9 g.

## Results

**Figure 3:**
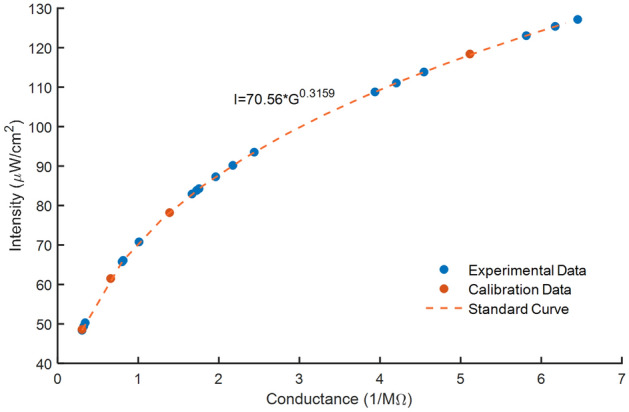
Intensity over conductance standard curve and experimental values.

Before UVC light intensity could be measured inside ARMOR, standard resistance values at set distances were recorded and a standard calibration curve was created (Fig. 3). A fabricated ZnO-based UV sensor quantitatively measures the level of UV intensity from the electrical resistance. The level of resistance of the sensor is inversely proportional to the level of UV intensity. This resistance response curve was used to calculate UV light intensity at different areas on the face covering. As expected in the calibration data, as sensor distance from the LED lamp increased, electrical resistance also increased and light intensity decreased. The plot of the standard curve revealed a nonlinear relationship between intensity and conductance, and the equation of the curve was used to calculate light intensity at different areas on the face covering from the experimental resistance measurements.

**Figure 4:**
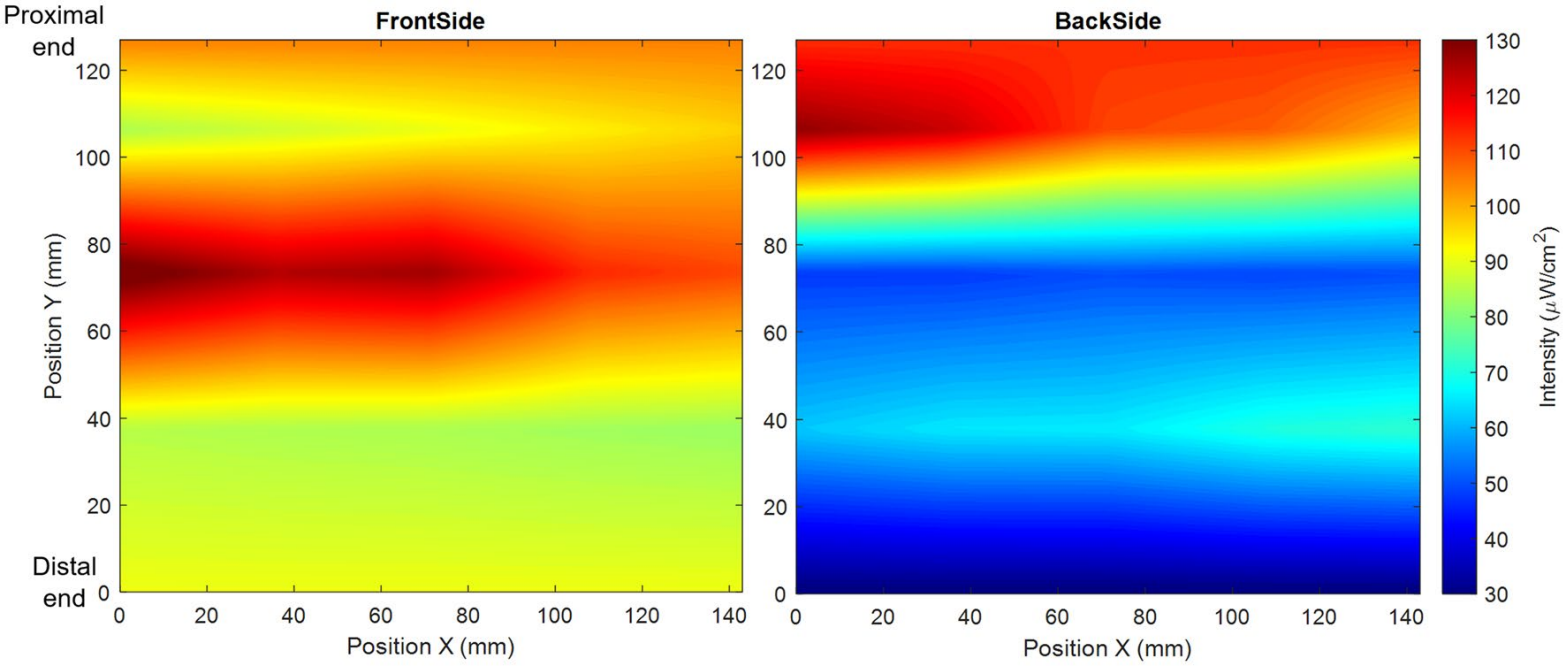
Contour plots of UVC intensity across front and back sides of the face covering. The “front” side faces the wearer’s environment, while the “back” side is in contact with the wearer’s nose and mouth. Areas of highest light intensity are shown in red, while areas of lower light intensity are shown in blue. The “proximal end” of the face covering refers to the top of the mask that sits on the bridge of the nose, and the “distal end” of the face covering refers to the bottom of the mask that is in contact with the neck area.

The “front” of the face covering, designated as the side exposed to the wearer’s environment, received UVC light at an intensity range of 82.92–127.16 μW/cm^2^ (Table 1, Fig. 4). It was found that sensor positions 4, 5, and 6 on the front side received the highest intensity light, which means that these areas of the mask are most directly exposed to the LED lamps. The “back” of the face covering is designated as the side in contact with the wearer’s nose and mouth. After experimentation, it was found that the light intensity on this side ranged from 48.39 to 123.05 μW/cm^2^ (Table 1). The similar upper range of intensity values from front to back suggests that there are areas on both surfaces that receive direct exposure to the UVC-producing LED lights. Sensor locations 1, 2, and 3 on the back of the mask received the lowest intensity UVC, which can be attributed to the distance from the LED lamps and the non-perpendicular angle to the incoming light the face covering rests at.

**Table 1:** Experimental resistance and calculated intensity values at 18 different locations on the face covering.

	Location on mask	Sensor position	Experimental resistance (MΩ)	1/R_e_	Calculated intensity (μW/cm^2^)
Front of face covering	Bottom	1	0.57	1.75	84.27
		2	0.58	1.72	83.81
		3	0.60	1.67	82.92
	Middle	4	0.162	6.17	125.40
		5	0.155	6.45	127.16
		6	0.220	4.55	113.84
	Top	7	0.51	1.96	87.29
		8	0.46	2.17	90.18
		9	0.41	2.44	93.52
Back of face covering	Bottom	1	1.25	0.80	65.76
		2	1.23	0.81	66.10
		3	0.99	1.01	70.79
	Middle	4	3.30	0.30	48.39
		5	2.92	0.34	50.30
		6	3.09	0.32	49.41
	Top	7	0.172	5.81	123.05
		8	0.238	4.20	111.05
		9	0.254	3.94	108.79

It was not feasible to place the sensor in some areas inside ARMOR. As an alternative, linear forecasting was used to extrapolate the intensity data to the edges of the face covering. After calculation, the area of least UVC exposure was determined to be the bottom edge of the back side of the mask, with a light intensity value of 29.06 μW/cm^2^. Given the expected areas of greatest and least exposure in the design, all intensity values calculated using the linear forecasting method were determined to be reasonable.

The exposure time required to inactivate different pathogens was calculated by dividing the given required energy dosage by the lowest experimentally determined UVC light intensity value (29.06 μW/cm^2^) (Table 2). Pathogens more susceptible to UVC radiation have a lower required energy dosage, and therefore take a shorter amount of time to become inactivated. *Streptococcus pyogenes*, the bacteria that causes the common illness “strep throat,” undergoes a 3-log reduction in under 42 s of exposure to the LED lamps inside ARMOR. A more UVC-resilient pathogen such as SARS-CoV-2 requires a longer exposure time to become inactivated, approximately 183 s to reach a 2-log reduction.

**Table 2:** Required energy dosages for inactivation and calculated exposure time values for seven common pathogens^15–17,24^.

Pathogen (% inactivation)	Required energy dosage (mJ/cm^2^)	Exposure time (s)
SARS-CoV-2 (99)	5.0	182.87
HCoV-229E (99.9)	1.7	71.14
HCoV-OC43 (99.9)	1.2	50.22
*Mycobacterium tuberculosis* (99.9)	3.3	113.54
H1N1 influenza (99.9)	3.8	130.75
*Escherichia coli* (99.9)	2.4	82.58
*Streptococcus pyogenes* (99.9)	1.2	41.29

Analysis of the experimental UVC intensity data revealed areas of the face covering with high exposure, and areas with less exposure. The contour plots of intensity reveal that the locations with the highest UV exposure are the center (y = 40–100 mm) and top (y = 110–127 mm) of the front side, and the top (y = 95–127 mm) of the back side of the face covering (Fig. 4).

## Discussion

ARMOR was designed to be an effective and elegant solution for minimizing face covering contamination and disposable mask waste. Using existing knowledge on the ability of ultraviolet C radiation to inactivate SARS-CoV-2 and other pathogens, the effectiveness of our design in sanitizing a reusable mask could be tested indirectly. The front side of the mask faces away from the wearer and is exposed the most to the environment. Therefore, it is advantageous that this side receives the highest intensity of the germicidal UVC light because it is likely the most easily contaminated. The back side of the mask faces the wearer, and it is important that this surface receives sufficient exposure during sanitization as well. The upper half of the back side is in contact with the nose and mouth of the user, so it is significant that this area receives a UV intensity greater than 70 μW/cm^2^. The bottom of the back side of the face covering is in contact with the wearer’s chin and neck area, which means that it likely receives the least amount of contamination. As a result, it is reasonable that this area receives a lesser intensity of the germicidal radiation.

It is important to note that regardless of the intensity in each location on the face covering, all areas on both sides are exposed to the UVC light. This means that even the areas of least intensity can reach the required energy dosage to be sanitized of different pathogens if the exposure time is sufficient. The exposure time was calculated by dividing the required energy dosage by the light intensity. This number was then recalculated for specific pathogens by dividing by the percentage of light energy produced by the 270 nm LED lamps used in ARMOR compared to the 254 nm light used to study SARS-CoV-2 and the 222 nm light used to study HCoV-229E and HCoV-OC43^15,16^. Limited research has been performed directly showing the ability of ultraviolet C radiation to inactivate SARS-CoV-2 and related coronaviruses, therefore it is unknown if longer wavelength, lower energy 270 nm light would take longer to have the same germicidal effects as the higher energy 222 nm and 254 nm lights. However, use of UVC radiation at 270 nm is a proven bactericidal and virucidal method^14^. The recalculated exposure times for HCoV-229E, HCoV-OC43, and SARS-CoV-2 therefore reflect the worst-case ability for the ARMOR prototype to inactivate more than 99.9% of these viruses. Some viruses such as SARS-CoV-2 and H1N1 Influenza require a higher UVC energy dosage to reach the same level of lethality. As a result, the exposure time can be extended accordingly to achieve broad-spectrum sterilization. The longest exposure time was determined to be at the bottom of the back side of the face covering, taking 183 s for SARS-CoV-2, the pathogen with the highest UVC resilience of those tested, to be 99% inactivated. Therefore, the 183 s is also the time ARMOR takes to sanitize all areas of the mask of all of the tested pathogens.

Overall, the high observed light intensity on both sides of the face covering and the relatively short time required to disinfect when compared to traditional washing methods indicates that we were successful in achieving our objective. The ARMOR prototype presents benefits to front-line healthcare workers by eliminating pathogens present on their masks, therefore reducing the spread of deadly infectious diseases. In between visiting patients, the mask can be removed and sanitized in just a couple of minutes, reducing the accumulation of bacteria and viruses on the face covering. Use of ARMOR instead of traditional disposable masks can also significantly reduce the amount of medical waste that ends up in landfills.

There are some limitations to the current design of ARMOR, though these can be addressed with future development of the design. The current version of the prototype has increased weight due to a large number of LED lamps and multiple batteries. However, both the size and weight can be reduced in future iterations with additional optimization. Given the short amount of time currently required to sanitize the face covering, the number of LED lamps can be reduced, eliminating the need for two batteries, and reducing the size and weight of ARMOR while still keeping the exposure time to a few minutes. Additionally, a cotton face covering was used in this version, which can allow infected water droplets to settle into the material and also acts as a poor filter for microbes. This could result in reduced exposure of pathogens to the UVC and decreased germicidal effects on the mask. As a solution, we intend to use nanofiber as an alternative to cotton in future iterations of our device. Nanofiber is already used as a common mask filter material, has a high filtration efficiency, and is UV resilient over long periods of time, making it the ideal material for use in ARMOR^22,23^. Our design of an Auto-sanitizing Retractable Mask Optimized for Reusability is effective at providing a lethal dose of ultraviolet-C radiation to the pathogens on the surface of a face covering, therefore protecting the wearer from infection and reducing unnecessary waste, while still leaving room for additional design improvement.

## Methods

Previous research has shown the effectiveness of UVC in inactivating a variety of pathogens, and the necessary energy dosages have been established^15–17,24^. The effectiveness of ARMOR was tested by measuring the UVC exposure at different locations on the surface of the face covering. Waveform Lighting provides specific details on their website on the irradiance (intensity) of a single 270 nm UVC-producing LED lamp at 1 in (2.54 cm) intervals up to 11 in (27.94 cm)^25^.

A fabricated ZnO-based UV sensor was used in the experiment to measure light intensity in terms of conductance, or the inverse of electrical resistance (G = 1/R)^26^. As shown by Pathak, Park, and Cho, the reliability and repeatability of the sensor used in our experiment has been demonstrated through repeated testing^26^. The screen-printing method is considered as an inexpensive, time-saving, and scalable process to fabricate electronic components^27^. In this process, the paste is squeezed through the screen and directly patterned on various substrates. The ZnO-based UV sensor fabrication involved screen-printing of silver interdigitated electrodes and a ZnO-based sensing layer on a flexible polyimide substrate. Silver paste was obtained from Daejoo Electronic Materials (Gyeonggi-Do, Korea) and ZnO-based paste was prepared using the hydrothermally synthesized ZnO nanoparticles, ethylene cellulose, ethanol, and terpinol. The sensors were cured at room temperature after printing.

In order to obtain measurements of UVC light intensity inside ARMOR, a standard curve of intensity versus conductance was first generated. To create the standard curve, the UV sensor was placed at fixed distances 2.54 cm, 5.08 cm, 7.62 cm, 10.16 cm away from a single LED lamp, and electrical resistance values at each distance were recorded and compared to the irradiance data from the LED manufacturer (Table 3).

**Table 3:** Given intensity and measured resistance of a single LED lamp at set distances from the UV sensor.

Distance (cm)	Intensity (μW/cm^2^)	Measured resistance (MΩ)	1/R_m_
2.54	118.4	0.20	5.12
5.08	78.2	0.72	1.39
7.62	61.5	1.52	0.66
10.16	48.6	3.30	0.30

After measuring the electrical resistance of the UV sensor when exposed to a single LED lamp at set distances, the irradiance data provided by Waveform Lighting could be used to create a standard curve of intensity over conductance. Once the experimental resistance values of the 18 different sensor locations on the mask inside the case were measured, the intensity of each location can be calculated using the standard curve. The y axis of the curve is intensity, and the x axis is conductance; therefore, the intensity of the UVC in μW/cm^2^ at each specific location is I = 70.536G^0^^.3159^. After a unit conversion from μW/cm^2^ to mW/cm^2^, the required energy dosage for each pathogen can be divided by the calculated intensity to determine the required exposure time to sanitize each location on the face covering. This exposure time was recalculated for the viruses SARS-CoV-2, HCoV-229E, and HCoV-OC43 using E = hc/λ, where E = light energy, h = Planck’s constant, c = speed of light, and λ = wavelength. This accounted for the difference in light energy between the referenced 254 nm (used in the study of UVC on SARS-CoV-2) and 222 nm UVC (used in the study of UVC on HCoV-229E and HCoV-OC43) compared to the 270 nm UVC used in ARMOR^15,16^.

**Figure 5:**
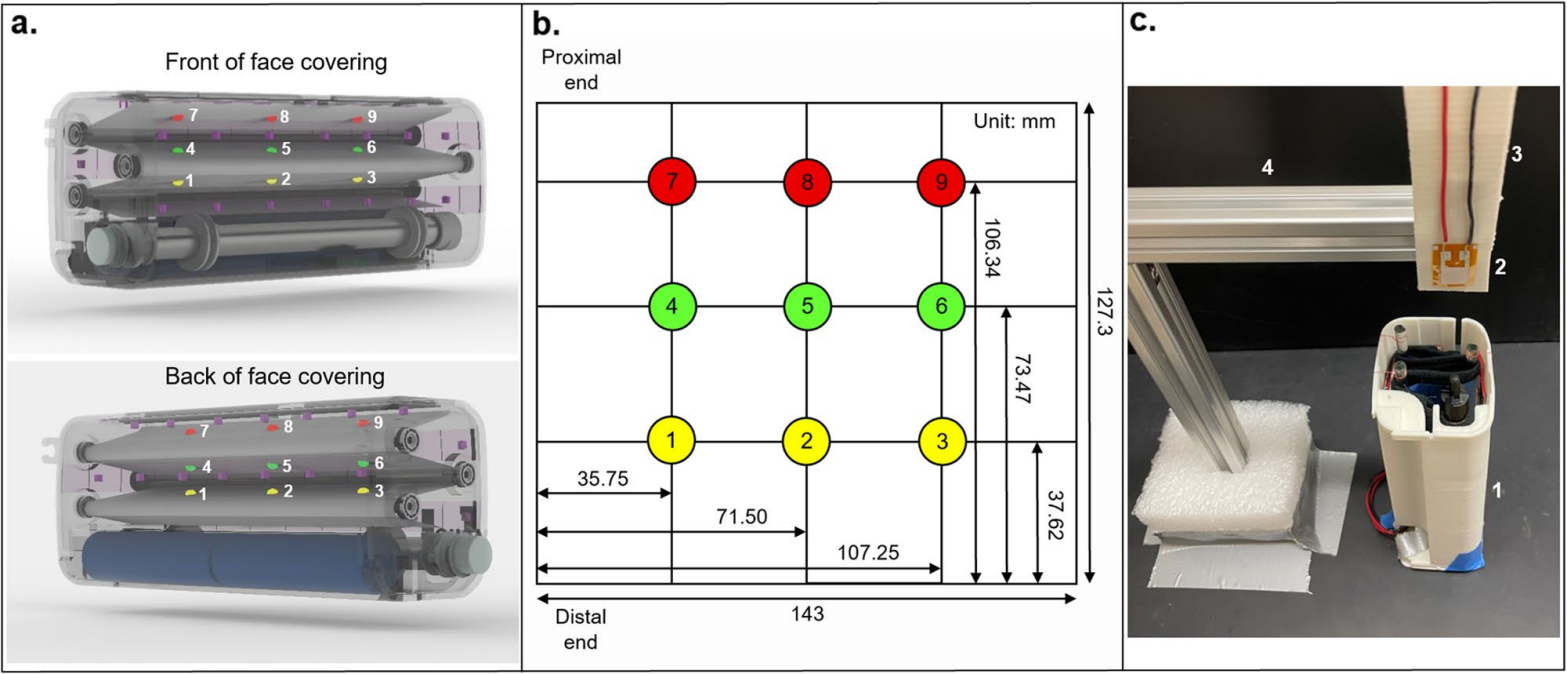
Locations of UV sensor placement on the face covering inside ARMOR (**a**), coordinate map of UV sensor placement on the face covering (**b**), and experimental setup using a ZnO-based UV sensor (**c**). The coordinate map and locations of sensor placement are numbered and color-coded by row, showing the precise locations of the UV sensor during testing relative to both the face covering and the other components of the device. Four components of the experimental setup are shown: the ARMOR prototype connected to a not shown external power supply (**1**), the Zn–O-based UV sensor (**2**), electrical wires running from the sensor to a not shown multimeter which recorded electrical resistance (**3**), and an adjustable tower clamp used to hold the sensor in place while resistance data was measured (**4**).

After the standard curve was generated, 9 sensor locations on both sides of the mask were selected in a 3X3 grid to measure UV light intensity inside the case (Fig. 5a,b). Using an adjustable clamp, the sensor was lowered into one of the selected locations on the face covering where it was held in place with the LED lamps turned on while electrical resistance was measured using a multimeter (Fig. 5c). This step was carried out for all 18 designated locations on the mask, and then the resistance values were compared to the standard curve to obtain the UV intensity data. Seven pathogens commonly found on face coverings were selected for analysis, and the exposure time to inactivate each was calculated by dividing the given required energy dosage by the lowest experimentally determined UVC light intensity value (29.06 μW/cm^2^).
